# Assessment of Vitamin K2 Levels in Osteoporotic Patients: A Case Control Study

**DOI:** 10.5539/gjhs.v6n6p82

**Published:** 2014-07-15

**Authors:** Akram Noori, Mahin Lashkari, Sonia Oveisi, Mohamad Reza Khair Khah, Ali Zargar

**Affiliations:** 1 Metabolic Diseases Research Center, Qazvin University of Medical Sciences, Qazvin, Iran

**Keywords:** bone densitometry, normal bone density, osteoporosis, vitamin K2

## Abstract

**Objective:**

The aim of this study was to measure the level of Vitamin K2 (Vit K2) in osteoporotic patients and individuals with normal bone density as controls.

**Materials and Methods:**

This case-control study was done in Outpatient Department of Rheumatology at Qazvin Boo-ali Sina Hospital in 2013. Participants were 50 patients with osteoporotic densitometry measured by DEXA (T score≤ -2.5) who were matched with 48 persons in control group with normal bone density (T score> -1). The level of Vit K2 in samples was measured using enzyme linked immunosorbent assay (ELISA). Data were analyzed by Mann-Whitney U test and Chi-square test.

**Results:**

The level of Vit K2 in patients with osteoporosis was not significantly different from the control group (

**Median:**

75.95 vs. 71.35 nmol/L, respectively; P-value: 0.709). The authors determined cut-offs 75 percentile of vitamin K2 in all participants that was 85 nmol/L and percentages of persons in two groups were similar.

**Conclusion:**

Although Vit K2 level in patients with osteoporosis was not significantly different from the control group, further studies are necessary to confirm the association of osteoporosis and Vit K2.

## 1. Introduction

Vitamin K (Vit K) is a fat soluble vitamin which is categorized to, phylloquinone (K1), menaquinones (K2) and Menadione (K3) (Plaza & Lamson, 2005). Phylloquinone (K1) family, also known as phytonadione based on relation to photosynthesis, is well known. The resources of Vit K1 are higher plants and green leafy vegetables (Shearer, 1995). Menaquinones (K2) produced naturally in a production series by intestinal bacteria and, not by higher plants (Plaza & Lamson, 2005).

Vit K deficiency is uncommon in healthy adults because: 1) Vit K is widespread in foods, 2) the Vit K cycle conserves it; and 3) bacteria in the large intestine usually synthesize menaquinones (Vitamin K2), but it is unclear whether significant amounts of produced Vit K2 are absorbed and utilized. The risk of Vit K deficiency is increased in patients who are taking Vit K antagonist like anticoagulant drugs, and individuals with significant liver damage or disease (Olson, 1999). As well, individuals with fat malabsorption disorders may be at higher risk of Vit K deficiency (Ferland, 2006). Exogenous Vit K is required for carboxylation of osteocalcin, which in turn allows osteocalcin to bind to hydroxyapatite mineral. A Vitamin K2 (Vit K2) preparation (menatetrenone) is widely used for treatment of osteoporosis in Japan (Rosen & Drezner, 2014). Observational data suggest that low vitamin K consumption or impaired Vit K status may be associated with an increased risk of fracture in elderly (Feskanich et al., 1999; Booth et al., 2004).

Seven clinical trials in Japan (primarily on postmenopausal women with osteoporosis) reported fracture data; and then used menaquinone for prevention of osteoporotic fractures. After administration of Vit K2, significant decrease was seen in vertebral, hip, and all non-vertebral fractures (Brar, 2010). It must be considered that these reports are only in Japanese women, who may have significant dietary differences from other countries (Brar, 2010). The aim of this study was to measure the levels of Vit K2 in individuals with normal bone density and osteoporotic patients.

## 2. Methods

In this case-control study, the osteoporotic women with low bone density (T-score ≤ 2.5 score) who were attending at outpatient rheumatology clinic of Boo-ali Sina hospital from June 2012 to March 2013 were compared with normal patients who were referred to this clinic for other problems unrelated to rheumatologic diseases. The control group had normal bone density during the same period. The study was approved by the ethics committee of Qazvin University of Medical Sciences. All participants signed the written informed consent and they could leave the study whenever wished.

The sample size was calculated using the following formula:


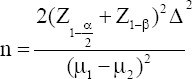


Considering α: 0.05, β: 20%, ∆: 200, µ_1_: 700 and µ_2_: 490 based on a pilot study by the authors, the calculated sample size was 40 for each group. This study was conducted in 50 osteoporotic patients and 48 healthy controls that were matched for age, gender, and socioeconomic status. Bone densitometry was done by DXA. In osteoporotic cases the range of T-score was found to be -2.5 to -4.1 in vertebra and -2.5 to -5.3 in femur while in normal cases the range of T-score was +1 to -0.9 in vertebra and femur.

**Table 1:** 

**T-Score Diagnosis**	Normal	0 to -1
	Osteopenic	-1 To -2.5
	Osteoporosis	≤ -2.5

Patients who were taking medications such as Aspirin, Cholestyramine, Phenytoin, or those who were taking broad-spectrum antibiotics for a long time as well as patients with liver disease and malabsorption were excluded from the study.

Two milliliters of peripheral venous blood were collected after 8 hour fasting from each participant. The measurement of Vit K2 was achieved by commercial ELISA kit based on its protocol (Glory science Co., Ltd: U.S.A.).

Kolmogorov Smirnov test was used to examine the normality of variables. Data were reported as median (minimum, maximum) or in number (percent). Variables were compared between the patients and the controls using Mann-Whitney U test or a Chi-square test. P-value < 0.05 was considered to be significant.

## 3. Results

In this case-control study, results of Vit K2 were compared in two groups. Fifty patients with osteoporotic bone density (T-score ≤ -2.5) and 48 age-matched persons as controls with normal bone density (T-score> -1) have attended. Table 1 has shown the demographic characteristics and Vit K2 level. Vit K2 level was not significantly different between two groups.

**Table 1:** Demographic characteristics and Vitamin K2 levels of participants in two groups

		Case group (N: 50)	Control group (N: 48)	P-value
**Gender** ^a^	Male	1(2.0)	3(6.2)	NS
	Female	49(98.0)	45(93.8)	

**Job** ^a^	Employment	8(16)	7(14.6)	NS
	Household	41(82)	40(83.4)	
	Retired	1(2)	1(2)	

**Marriage Status** ^a^	Single	4(8)	6(12.5)	NS
	Married	46(92)	42(87.5)	

**Age** ^a^	30-49	3(6)	7(14.6)	NS
	50-69	43(86)	39(81.2)	
	≥70	4(8)	2(4.2)	

**BMI** ^a^	<25	14(31.1)	8(19.5)	NS
	25-30	18(40)	15(36.6)	
	>30	13(28.9)	18(43.9)	

**Education** ^a^	Uneducated	12(24)	9(18.8)	NS
	<12 years	25(50)	28(58.3)	
	>12 years	13(26)	11(22.9)	

**Vit K2** ^b^		75.95 (26.8-341.1)	71.35 (37.8- 498.6)	NS

a data are presented as number (percent);

b Data are presented as median (min - max); NS: not significant.

In addition, the authors determined cut-offs 75 percentile of vitamin K2 in all participants that was 85 nmol/L. Table 2 demonstrates that the same as previous table, percentages of persons in two groups were similar. Furthermore, we got interested to analyze Vit K2 in two groups based on categorized BMI and age. Table 3 shows that the level of Vit K2 in BMI of 25-30 was more than the others, however, this difference was not statistically significant between two groups.

**Table 2:** Comparison of participants based on the cut-offs 75 percentile

	Case group N (%)	Control group N (%)	P -value
**Vit K2 < 85**	39(78.0)	36 (75.0)	0.813

**Vit K2 > 85**	11(22.0)	12(25.0)	

**Table 3:** Comparison of vitamin K2 in two groups based on categorized BMI and age

				Median	Min	Max	P-value
**Vit K2**	**BMI**	<25	Case	75.95	52.9	341.1	0.539
			Control	72.15	50.2	498.6	

		25-30	Case	82.1	26.8	314.3	0.563
			Control	84.4	58.0	305.7	

		>30	Case	73.1	48.2	171.5	0.734
			Control	71.2	48.2	439.5	

	**Age**	30-49	Case	66.0	48.8	72.9	0.206
			Control	77.1	43.3	498.6	

		50-69	Case	77.0	26.8	341.1	0.726
			Control	72.1	37.8	439.5	

		≥70	Case	83.1	63.0	111.5	0.165
			Control	56.6	48.2	65.0	

## 4. Discussion

Like other fat-soluble vitamins, absorption of Vit K depends on bile salts, so the clinical assessment of Vit K is important in patients with malabsorption syndromes and liver diseases. Assessment of Vit K in plasma is difficult because it is in nanosmolar concentrations (Thane, 2002; McKeown et al., 2002; Booth et al., 1997). Large differences in average levels of Vit K have been reported in different populations which explain the range of 0.22 to 8.88 nmol/L. (Azharuddin, O’Reilly, Gray, & Talwar, 2007). Recently several methods for direct and accurate measurement of all types of Vit K in blood circulation have been developed (Suhara, Kamao, Tsugawa, & Okano, 2005; Booth, Lichtenstein, & Dallal, 2002). Vit K acts as a cofactor for carboxylase enzyme. This allows the protein with high affinity for calcium ions to bind to osteocalcin. Vit K dependent carboxylation allows proteins to bind to hydroxyapatite (Hoang, Sicheri, Howard, & Yang, 2003).

In recent years, many studies about Vit K2 and its role in osteoporosis were taken. Several Japenese studies showed that Vit K2 in patients with osteoporosis is low and giving Vit K2 to them increases bone density (Rosen & Drezner, 2014). Several studies in different countries have been performed about the effect of Vit K2 in osteoporotic patients and its effect on bone density. In some of these studies, it is suggested that Vit K2 has positive impact on osteoporosis, but others shows no effect (Rosen & Drezner, 2014; Feskanich, et al., 1999; Booth, et al., 2004; Cockayne et al., 2006). Observational data indicate that Vit K deficiency may be associated with an increased risk of fracture in older men and women (Rosen & Drezner, 2014; Feskanich et al., 1999; Booth et al., 2004). In our study; there is no significant difference in level of Vit K2 in osteoporotic patients than normal group.

Vitamin K2 plays a role in bone formation. There are many studies on the effect of vitamin K2 on bone density and risk of bone fracture. But none of the recent studies assess the Vit K level in the serum of patients. Therefore we measured the serum level of Vit K2 in 50 osteoporotic patients and in 48 individuals with normal bone density. There was no significant difference between two groups. Also there was no significant difference in serum Vit K2 level in postmenopausal and nonmenopausal women.

Vitamin K1 is found in leafy green vegetables particularly at high levels in plants but is found at low levels in meat and cheese and bread. Vit K2 is found in the meat, vegetable oils and cheeses. The food intakes of Vit K are high in western countries but not so in Japan (Booth, Pennington, & Sadowski, 1996).

In a study performed on 381 menopausal women in North America, they were given Vit K2 or placebo for a period of 12 months. In the group who received Vit K2 no effect was found on BMD of the lumbar vertebra and femur (Binkley et al., 2009). Vitamin K2 is used extensively in Japan for the treatment of osteoporosis (Rosen & Drezner, 2014). Many studies about the efficacy of Vit K2 to increase the bone density were reported from Japan which are especially nutritional ones (Bolton-Smith et al., 2007). Therefore, those studies should be interpreted with caution. In a double–blind placebo-controlled study on nonosteoporotic postmenopausal women in North America, the aim was to evaluate the Vit K2 treatment on markers of skeletal turnover and BMD which showed no effect on BMD (Rosen & Drezner, 2014; Binkley et al., 2009; Booth et al., 1996). In a 3-yr double-blind study of 452 men and women between the age 60 to 80 years, they received a multivitamin which contained either 500 μg/d or no phylloquinone plus a daily calcium (600 mg elemental calcium) and vitamin D (400 IU) supplement and the treatment did not make any change in BMD of anatomical sites (Booth et al., 2008). In a study of postmenopausal Korean women, 78 postmenopausal women older than sixty years over the past 6 years were given Vit K along with vitamin D and calcium, which resulted in decreased serum level of undercarboxylated osteocalcin and led to increased bone mineral density (Je et al., 2011). Although Vit K2 level in patients with osteoporosis was not significantly different from the control group in the present study, further studies are necessary to confirm the association of osteoporosis and Vit K2.
